# Mapping of spastic muscle activity after stroke: difference between passive stretch and active contraction

**DOI:** 10.1186/s12984-024-01376-z

**Published:** 2024-06-14

**Authors:** Tian Xie, Yan Leng, Pan Xu, Le Li, Rong Song

**Affiliations:** 1 Key Laboratory of Sensing Technology and Biomedical Instrument of Guangdong Province, School of Biomedical Engineering, Sun Yat-sen University, Shenzhen, 518107 China; 2 Shenzhen Research Institute of Sun Yat-sen University, Sun Yat-sen University, Shenzhen, 518107 China; 3 Department of Rehabilitation Medicine, The First Affiliated Hospital of Sun Yat-sen University, Sun Yat-sen University, Guangzhou, 510080 China; 4 Institute of Medical Research, Northwestern Polytechnical University, Xi’an, 710072 China

**Keywords:** Spasticity, HD-sEMG, Spatial heterogeneity, Stretch reflex, Voluntary movement

## Abstract

**Background:**

Investigating the spatial distribution of muscle activity would facilitate understanding the underlying mechanism of spasticity. The purpose of this study is to investigate the characteristics of spastic muscles during passive stretch and active contraction by high-density surface electromyography (HD-sEMG).

**Methods:**

Fourteen spastic hemiparetic subjects and ten healthy subjects were recruited. The biceps brachii (BB) muscle activity of each subject was recorded by HD-sEMG during passive stretch at four stretch velocities (10, 60, 120, 180˚/s) and active contraction at three submaximal contraction levels (20, 50, 80%MVC). The intensity and spatial distribution of the BB activity were compared by the means of two-way analysis of variance, independent sample *t*-test, and paired sample *t*-test.

**Results:**

Compared with healthy subjects, spastic hemiparetic subjects showed significantly higher intensity with velocity-dependent heterogeneous activation during passive stretch and more lateral and proximal activation distribution during active contraction. In addition, spastic hemiparetic subjects displayed almost non-overlapping activation areas during passive stretch and active contraction. The activation distribution of passive stretch was more distal when compared with the active contraction.

**Conclusions:**

These alterations of the BB activity could be the consequence of deficits in the descending central control after stroke. The complementary spatial distribution of spastic BB activity reflected their opposite motor units (MUs) recruitment patterns between passive stretch and active contraction. This HD-sEMG study provides new neurophysiological evidence for the spatial relationship of spastic BB activity between passive stretch and active contraction, advancing our knowledge on the mechanism of spasticity.

**Trial registration:**

ChiCTR2000032245.

## Background

Spasticity is one of the disabling upper motor neuron syndromes following central nervous system (CNS) lesions, which is characterized by a velocity-dependent increase in muscle tone resulting from hyperexcitability of stretch reflex [1]. Studies have shown that the incidence of spasticity is as high as 40% after stroke [2, 3]. It can severely affect the activity of daily living (ADL) and quality of life (QoL) of stroke survivors through various secondary complications [4, 5]. A clear understanding of spasticity may facilitate the development of its interventions, which is important in preventing the onset of spasticity or slowing or limiting its progression [6]. The hyperexcitability of the alpha motoneuron pool is thought to play a critical role in the mechanism of spasticity [7–9].

Spasticity is involuntary muscle activity due to diminished regulation of inhibitory reflex pathways [10, 11]. In fact, the loss of descending corticospinal pathways to spinal motoneurons after stroke not only affects reflex excitability but also voluntary movement [12, 13]. The relationship of abnormal muscle activity between spasticity and voluntary movement disorders in stroke survivors still remains unclear [14, 15]. However, the characteristics of spastic muscles during voluntary movement have not been fully understood in the study of spasticity to date. Although different from traditional passive methods, it is essential to assess the response of spastic muscles during voluntary movement [16].

Despite the fact that clinical scales are widely used to evaluate spasticity, several studies have demonstrated their lack of validity and reliability in quantifying the involuntary muscle activity [17, 18]. To address this challenge, the surface electromyography (sEMG) technique was used to assess the reflex muscle response to passive stretch [19–21]. However, the conventional sEMG technique limits the sufficient and accurate recording of neuromuscular function [22, 23]. Not restricted to the number of electrodes, the high-density sEMG (HD-sEMG) technique has the potential to explore the spatial distribution of muscle activity through a closely spaced two-dimensional electrode grid, opening new possibilities for exploring the neuromuscular function [24, 25]. Recent studies based on HD-sEMG have demonstrated that muscle activity is spatially heterogeneous, which is associated with the non-uniform distribution and recruitment of motor units (MUs) within the muscle [26, 27]. Comprehensive knowledge of neuromuscular dysfunction associated with spasticity is necessary to further understand its underlying mechanism.

In this study, the HD-sEMG technique was applied to record the biceps brachii (BB) muscle activity of healthy subjects and spastic hemiparetic subjects during passive stretch and active contraction. We hypothesized that the spastic hemiparetic subjects would exhibit heterogeneous activation caused by an excessive response to muscle stretch. We further hypothesized there would be an association between the spatial heterogeneity of spastic BB activity during passive stretch and active contraction.

## Methods

### Participants

Fourteen spastic hemiparetic subjects (12 male; 47.5 ± 12.1 years) and ten healthy subjects (6 male; 24.3 ± 1.7 years) volunteered to participate in the study. They were all right-handed. The hemiparetic subjects were selected based on the following criteria: (1) hemiplegia secondary to the first ischemic or hemorrhage stroke; (2) modified Ashworth scale (MAS) scored 1–2 for the elbow flexors on the affected side; (3) manual muscle test (MMT) scored greater than or equal to 3 for the elbow flexors on the affected side; (4) not on medication for spasticity; (5) ability to follow instructions and sign an informed consent. Table 1 showed the basic characteristics of all hemiparetic subjects. This study was approved by the Ethics Committee of the First Affiliated Hospital of Sun Yat-sen University and registered at the Chinese Clinical Trial Registry (ChiCTR2000032245). All procedures were conducted according to the Declaration of Helsinki. All of the subjects enrolled in this study signed the informed consent before the experiment procedures.

**Table 1:** Basic characteristics of hemiparetic subjects

Patient	Sex	Age (yo)	Height (cm)	Weight (kg)	Months since stroke	Affected side	Modified Ashworth score	Muscle strength score
1	Male	34	172	74	3	Left	1	3
2	Male	28	180	87	19	Right	2	4
3	Male	51	165	65	17	Left	1＋	4
4	Female	57	155	45	4	Right	1＋	3
5	Male	44	164	60	3	Right	1	3
6	Male	56	180	79	1	Right	1	4
7	Male	59	165	57	3	Right	1＋	3
8	Female	54	160	60	28	Left	1	4
9	Male	71	170	60	1	Right	1	3
10	Male	53	178	92	1	Right	1	3
11	Male	50	173	80	3	Left	2	3
12	Male	27	170	60	4	Right	1	4
13	Male	43	171	68	4	Right	1＋	4
14	Male	38	170	65	9	Left	1＋	4

### Experimental protocols

**Fig. 1:**
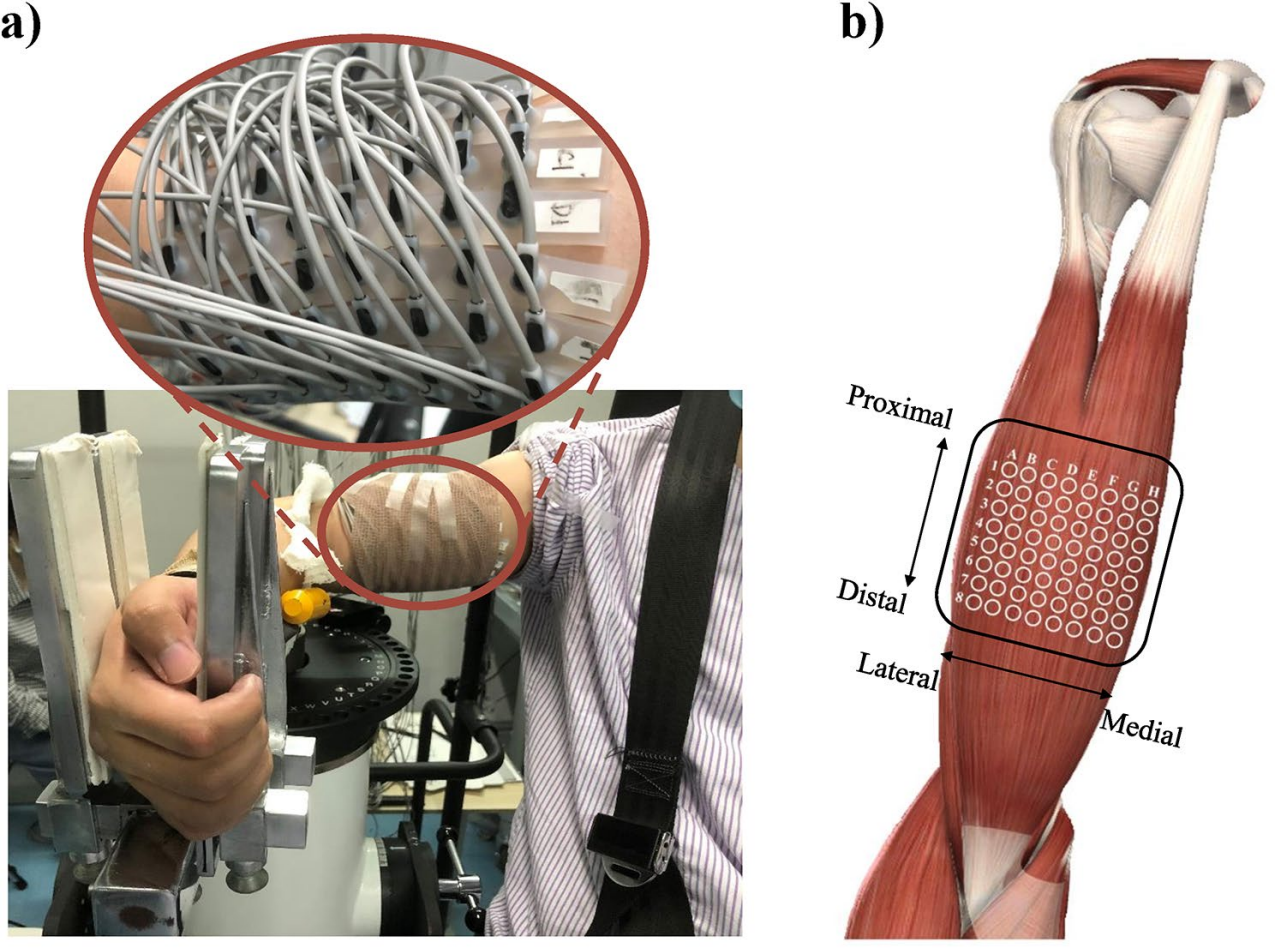
Illustration of the experimental setup. **a** The initial position of subjects and the surface EMG electrode arrangement. **b** The relative position of the 8 × 8 electrode array to the body

The subjects sat in a comfortable posture on the seat of the isokinetic dynamometer system (CSMi, HUMACNORM, USA), and the axis of motion of their elbow joint was aligned with the axis of rotation of the isokinetic dynamometer. The dominant or hemiparetic forearm and wrist of healthy or hemiparetic subjects were fixed in a neutral position on the anti-gravity customized apparatus, and the trunk was stabilized by straps. Figure 1a showed the initial position of subjects with shoulder abduction and elbow flexion at about 90 degrees. Following familiarization with the whole experimental procedure, the subjects completed the passive and active tasks on the isokinetic dynamometer system.

*Passive task.* In the passive task, the subjects were instructed to relax completely to perform passive movement of the elbow joint at four different velocities. The velocity sequence was 10, 60, 120, and 180°/s with a 60 s break between each velocity, and 3 trials were performed in each velocity with an interval of 10 s. A trial consisted of two consecutive back and forth movements from the onset position of elbow flexion at 90 degrees to the end position of maximum elbow extension. The process of elbow extension was defined as the passive stretch of the BB, and the data of the first extension process was extracted for subsequent analysis.

*Active task.* In the active task, the maximum voluntary contraction (MVC) of individual subjects was first measured when the subjects performed three maximum isometric elbow flexions at 90 degrees. Verbal encouragement was provided to ensure maximum contraction. The largest force value of the 3 trials was defined as the MVC amplitude. Then the MVC amplitude of each subject was used to define three submaximal contraction levels respectively. The subjects were instructed to perform isometric elbow flexion for 7 s at a predefined target level in the order of 20%, 50%, and 80%MVC. The real-time visual feedback of force output was displayed on the computer monitor by a green diamond cursor. 3 trials were performed for each submaximal contraction level with 60 s of rest in between to minimize fatigue.

### HD-sEMG Acquisition

During both passive and active tasks, HD-sEMG signals from the BB of the dominant arm and the hemiparetic arm were collected in monopolar mode by the REFA system (TMSi, REFA, the Netherlands, sampling frequency 2,000 Hz). As shown in Fig. 1, the electrode array organized in 8 rows by 8 columns, consisting of 64 independent channels with a 2 mm single electrode diameter and a 12 mm inter-electrode distance in both directions. Before placing the electrode array, the position of the BB was determined by palpation. The columns of the electrode array were arranged along the direction of the muscle fibers, and the center of the electrode array was placed on the BB recommended by SENIAM. The reference electrode was placed on the styloid process of the ulna of the tested arm. To prevent displacement, the electrode array and reference electrode were tightly secured by an elastic bandage. Moreover, the surface of the electrodes was filled with conductive gel, and the skin over the belly of the BB was abraded with abrasive paste and cleaned with alcohol.

### HD-sEMG analysis

Acquired HD-sEMG signals were processed offline using MATLAB software (R2020a, The MathWorks Inc., MA, USA). The signals were notch filtered at 50 Hz to remove power line interference and bandpass filtered at 20–500 Hz with a 6th-order Butterworth bandpass filter to reduce motion artifacts and noise. Through visual inspection, signals with low signal quality caused by poor electrode-skin contact were excluded and replaced by the linear interpolation of adjacent channels. Passive stretch epochs of different lengths were extracted from the signals during the passive task according to different stretch velocities, and 3 s stable active contraction epochs of all submaximal contraction levels were extracted from the signals during the active task. The epoch with the maximum number of effective channels was selected from three trials of each task to compute the root mean square (RMS) value on the 200ms non-overlapping window and averaged over all windows for each channel:

1 $$RMS=\frac{1}{M}\sum _{m=1}^{M}\sqrt{\frac{1}{N}\sum _{n=1}^{N}{u}^{2}\left(n\right)}$$

where *u* is an sEMG signal for each channel, *N* is the total number of samples in the 200ms window length, *M* is the total number of segments of the selected HD-sEMG epoch divided by 200ms window. To highlight the spatial distribution of activation patterns and compare muscle activity among different tasks and subjects, the maximum RMS value (${RMS}_{max}$) of the *M* segments in the three MVCs of each subject is used for their normalization respectively. Then the activation map of the BB based on the normalized RMS (nRMS) values obtained from the 8 × 8 electrode array was constructed:

2 $${AM}_{i,j}={nRMS}_{i,j}=\frac{{RMS}_{i,j}}{{RMS}_{max}}$$

where ${RMS}_{i,j}$ is the RMS value of the channel located in the *i*-th row and *j*-th column of the electrode array. The amplitude of each pixel in the map represents the normalized activation intensity of the BB, so the map can be considered as three dimensions where the altitude in the topographical map is given by ${AM}_{i,j}$. In the nRMS activation maps, blue and yellow pixels indicated low and high BB activity, respectively. For graphical representation, the cubic spline interpolation algorithm was used for the nRMS value, but only the original value was used in data and statistical analysis. The data of healthy subjects and spastic hemiparetic subjects were averaged as the healthy group and spastic group for comparison.

To quantify the global BB activity, the average intensity of the nRMS activation maps across all 64 channels was calculated. To describe the spatial distribution of the BB activity across the electrode grid, the center of gravity (CoG) of the nRMS activation maps was extracted [25]:

3 $$CoG\left(i,j\right)= \frac{1}{\sum _{i}\sum _{j}{AM}_{i,j}}\sum _{i,j}{AM}_{i,j }. \left[\begin{array}{c}i\\ j\end{array}\right]$$

where ${AM}_{i,j}$ represented the nRMS activation maps while *i* and *j* are the row and column of the electrode array, respectively. Thus, obtained *i*- and *j*- coordinates of the CoG are used to represent the lateral-medial and proximal-distal directions of the muscle. Since the passive stretch of 10°/s was recorded from the resting state without inducing muscle activation [28], the nRMS activation maps used to calculate the CoG were all subtracted from it for each subject. The data was further divided into the passive stretch at three stretch velocities and the active contraction at three contraction levels.

Furthermore, the nRMS activation maps were segmented to identify the location of the activation area to calculate the area overlapping. In this study, an h-dome transform ${D}_{h}$*(AM)* was applied over the image *AM*, and a morphological opening (*γ*) was applied to the resulting image ${D}_{h}$ [27]. When more than one cluster was identified, only the channels within the cluster with the highest nRMS value were considered as the relevant channels [29]. Figure 4a showed an example of segmentation with the relevant channels indicated by black dots. Afterward, the overlapping degree of the identified activation area was calculated as the intersection of the clusters, which is the number of relevant channels common to each pair of clusters. The number of identified relevant channels in the largest activation area of the two clusters was used to normalize the overlapping degree [30]. After segmenting the nRMS activation maps of individual tasks, two different overlapping degrees of the identified activation area were calculated. The first was the ‘within-task’ overlapping degree. Specifically, for a given subject, the overlapping degree was calculated between all possible stretch velocity pairs or between all possible contraction level pairs. As illustrated in Fig. 4b, the average ‘within-task’ overlapping degree identified from the nRMS activation maps of the three stretch velocities for the spastic group (82.3% ± 14.3) and the three contraction levels for the healthy group and spastic group (84.7% ± 10.0; 83.5% ± 14.6) were all higher than 80%. In order to quantitatively compare the BB activity distribution activated through the two tasks, the data of three stretch velocities and three contraction levels were averaged as the passive stretch and active contraction, respectively. The second was the ‘between-tasks’ overlapping degree, which was calculated between tasks that belonged to passive stretch and active contraction.

### Statistical analysis

All statistical analyses were performed in the software IBM SPSS Statistics version 21 (SPSS Inc., IL, USA). Data summarized in the text and figure are presented as mean ± SD. Statistical significance level was set at *p* < 0.05 for all tests. In the comparison of global BB activity, the two-way analysis of variance (ANOVA) with repeated measures was applied to assess the effects of the group (healthy group and spastic group) and task (four stretch velocities and three contraction levels separately) on the average intensity. In all cases, significant differences revealed by two-way ANOVA were followed by the post hoc independent sample *t*-test to compare the differences between the two groups at the same task and one-way ANOVA to compare the differences among different tasks within the same group. Since the healthy group did not show a significant increase in reflex activity, only the data on the spatial distribution of BB activity in the spastic group were considered during passive stretch. In order to test whether the spatial distribution of the spastic BB had changed during active contraction, the independent sample *t*-test was conducted to analyze the *i*- and *j*- coordinates of the CoG. In the comparison of the spatial distribution of BB activity during passive stretch and active contraction within the spastic group, the paired sample *t*-test was conducted to analyze the *i*- and *j*- coordinates of the CoG.

## Results

### Spastic group VS. healthy group

**Fig. 2:**
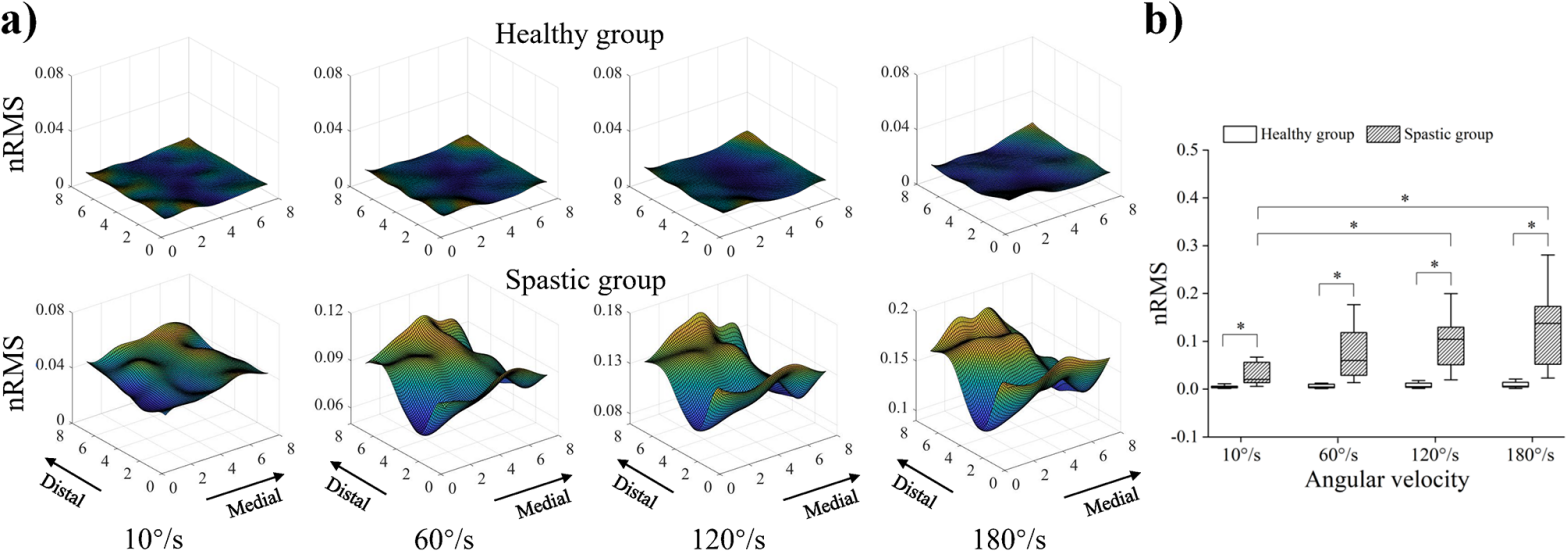
Normalized root mean square (nRMS) results at four stretch velocities during the passive task. **a** Topographic maps constructed from nRMS values of the healthy group and spastic group. The nRMS activation map was up-sampled from 8 × 8 to 71 × 71 through cubic spline interpolation to obtain a sufficient resolution for visualization. The amplitude of each pixel in the map represents the normalized activation intensity of the biceps. The yellow color refers to the maximum activation of the channel while the blue color refers to the minimum activation of the channel. **b** The average intensity of the nRMS activation maps for the healthy group and spastic group. Boxes indicate interquartile range, whiskers indicate 1.5× inter-quartile values, solid horizontal lines in the box indicate the median, and asterisks indicate the significant difference (*p* < 0.05)

Figure 2 presents the nRMS activation maps (Fig. 2a), and the average intensity of the nRMS activation maps (Fig. 2b) of the healthy group and spastic group at four stretch velocities during the passive task. As the velocity increased, the spastic BB exhibited heterogeneous activation while the healthy BB had no obvious activation. The two-way ANOVA analysis showed that the group (*F* = 44.112, *p* = 0.000) and task (*F* = 3.249, *p* = 0.026) had a significant effect on the average intensity. The post hoc analysis of the independent sample *t*-test indicated that the average intensity of the spastic group was significantly higher than the healthy group at 10˚/s, 60˚/s, 120˚/s, and 180˚/s (*p* = 0.013, *p* = 0.002, *p* = 0.001, *p* = 0.000). The post hoc analysis of the one-way ANOVA in the spastic group showed that the average intensity of 120˚/s and 180˚/s was significantly higher than that of 10˚/s (*p* = 0.015, *p* = 0.002), as shown in Table 2.

**Table 2:** Average intensity of the nRMS activation maps

		Healthy group	Spastic group
Passive task	10°/s	0.01 ± 0.00^*^	0.04 ± 0.04^+^
	60°/s	0.01 ± 0.00^*^	0.08 ± 0.07
	120°/s	0.01 ± 0.01^*^	0.12 ± 0.09^+^
	180°/s	0.01 ± 0.01^*^	0.15 ± 0.10^+^
Active task	20%MVC	0.06 ± 0.02^*+^	0.17 ± 0.06^+^
	50%MVC	0.18 ± 0.05^*+^	0.27 ± 0.08^+^
	80%MVC	0.34 ± 0.08^+^	0.35 ± 0.10^+^

^*^ Significantly difference (*p* < 0.05) between the healthy group and spastic group

^+^ Significantly difference (*p* < 0.05) within the healthy group or spastic group

**Fig. 3:**
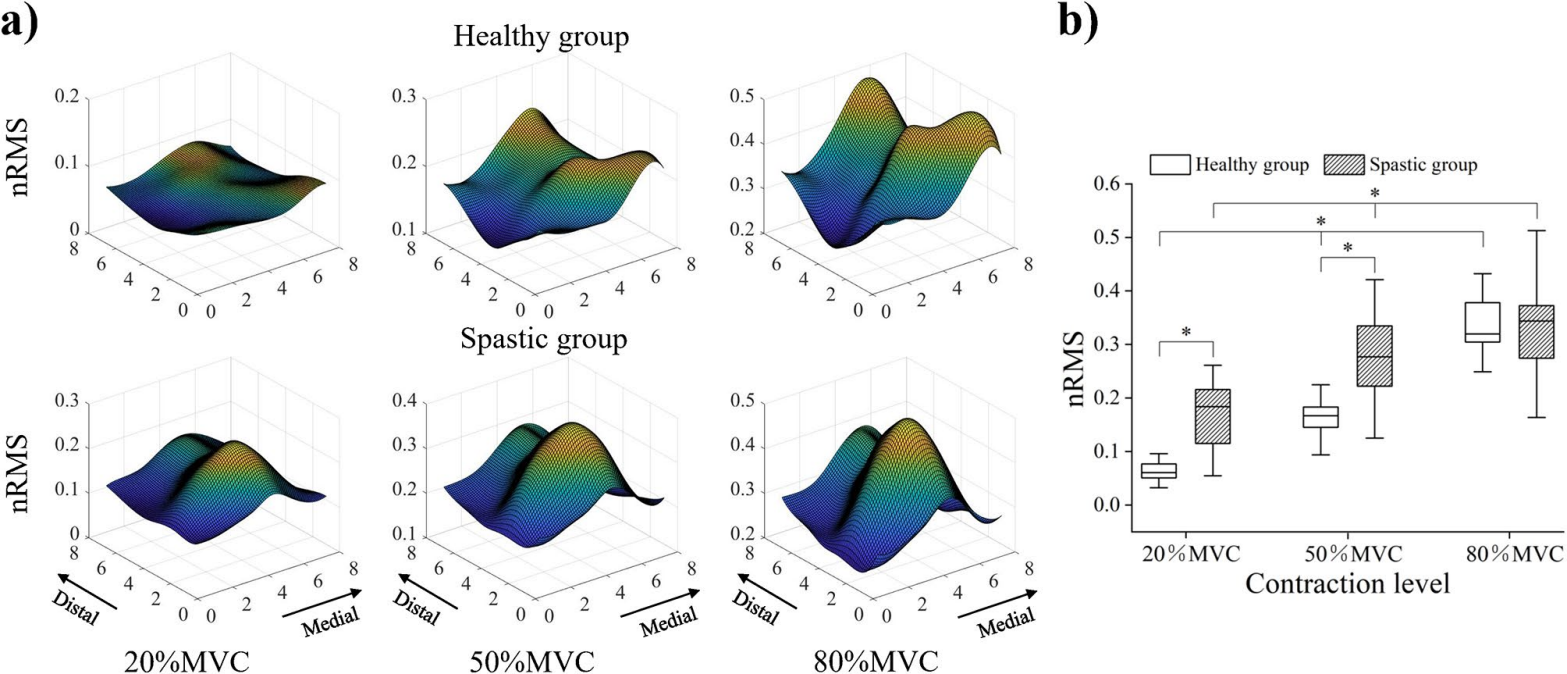
Normalized root mean square (nRMS) results at three contraction levels during the active task. **a** Topographic maps constructed from nRMS values of the healthy group and spastic group. The nRMS activation map was up-sampled from 8 × 8 to 71 × 71 through cubic spline interpolation to obtain a sufficient resolution for visualization. The amplitude of each pixel in the map represents the normalized activation intensity of the biceps. The yellow color refers to the maximum activation of the channel while the blue color refers to the minimum activation of the channel. **b** The average intensity of the nRMS activation maps for the healthy group and spastic group. Boxes indicate interquartile range, whiskers indicate 1.5× inter-quartile values, solid horizontal lines in the box indicate the median, and asterisks indicate the significant difference (*p* < 0.05)

**Fig. 4:**
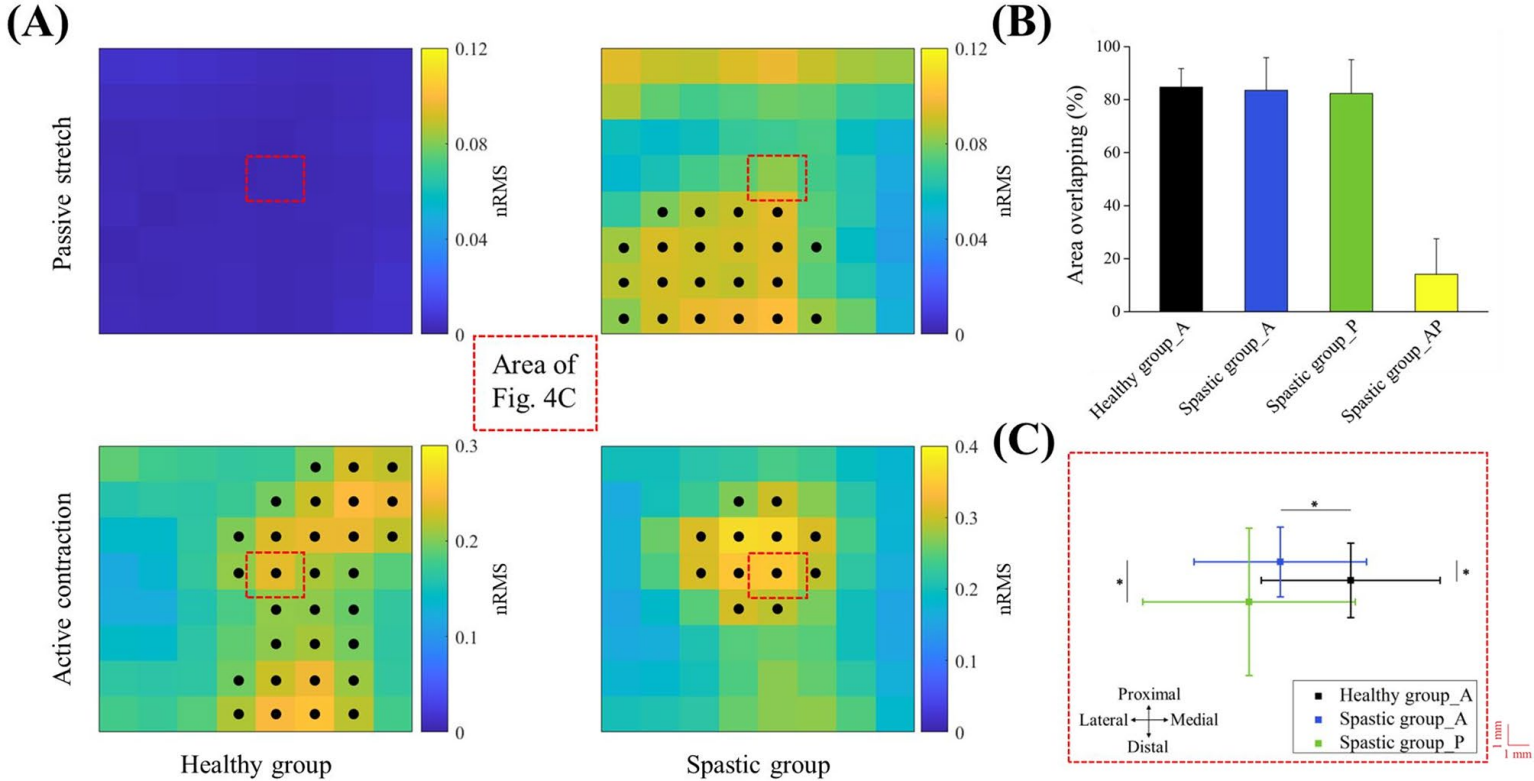
Segmentation example, degree of area overlapping and mean center of gravity (CoG) results. **a** Average nRMS activation maps of passive stretch and active contraction and their segmented results for the healthy group and spastic group. Final segmented relevant channels are indicated by black dots. The red dotted lines indicate the area where the mean CoGs are plotted. **b** The overlapping degree of the identified activation areas during passive stretch and active contraction. The area overlapping of Healthy group_A and Spastic group_A represent the average ‘within-tasks’ overlapping degree between all possible contraction level pairs in the healthy group and spastic group, respectively. The area overlapping of Spastic group_P represents the average ‘within-task’ overlapping degree between all possible stretch velocity pairs in the spastic group. The area overlapping of Spastic group_AP represents the ‘between-tasks’ overlapping degree between passive stretch and active contraction in the spastic group. Error bars designate standard deviation of mean. **c** Mean CoG position of the nRMS activation maps during passive stretch and active contraction. The black symbol and blue symbol are used for the average CoG during active contraction in the healthy group and spastic group, respectively. The green symbol is used for the average CoG during passive stretch in the spastic group. Error bars designate standard deviation of mean. Statistical differences (*p* < 0.05) between CoGs in the lateral-medial direction or the proximal–distal direction are marked with horizontal or vertical lines, respectively

Figure 3 presents the average nRMS activation maps (Fig. 3a), and the average intensity of the nRMS activation maps (Fig. 3b) of the healthy group and spastic group at three contraction levels during the active task. It could be seen that the higher nRMS values of the healthy group were scattered in the proximal and distal directions, while those of the spastic group were mainly clustered in the proximal direction. Specifically, there were two EMG peaks for the healthy group, whereas only one EMG peak was observed in the spastic group. The two-way ANOVA analysis showed a significant effect of the group (*F* = 14.564, *p* = 0.000) and task (*F* = 52.844, *p* = 0.000) on the average intensity. The post hoc analysis of the independent sample *t*-test indicated that the average intensity of the spastic group was significantly higher than the healthy group at 20%MVC and 50%MVC (*p* = 0.000, *p* = 0.004). The post hoc analysis of the one-way ANOVA of both the healthy and spastic groups showed that the average intensity of 50%MVC and 80%MVC was significantly higher than that of 20%MVC (*p* = 0.000, *p* = 0.000; *p* = 0.003, *p* = 0.000), and the average intensity of 80%MVC was also significantly higher than that of 50%MVC (*p* = 0.000; *p* = 0.034). For the spatial distribution of BB activity during active contraction, the independent sample *t*-test showed that the *i*- and *j*- coordinates of the CoG in the spastic group were smaller than the healthy group (4.51 ± 0.29 vs. 4.74 ± 0.30, *p* = 0.001; 4.45 ± 0.11 vs. 4.51 ± 0.12, *p* = 0.037), which suggested a lateral and proximal shift of its CoG (Fig. 4b).

### Passive stretch VS. active contraction

Figure 4 illustrates the average nRMS activation maps and their segmentation examples (Fig. 4a), and the overlapping degree of the identified areas (Fig. 4b), and the mean CoG position of the nRMS activation maps (Fig. 4c) during passive stretch and active contraction in the spastic group and healthy group. The red dotted lines indicate the area where the mean CoGs are plotted. It could be observed that in the spastic group, most of the activation area of the passive stretch did not overlap with the active contraction. Figure 4b confirmed the result: the area overlapping between passive stretch and active contraction in the spastic group was only 14.1% ± 13.4. Comparison of the CoG in the spastic group by the paired sample *t*-test also showed that the passive stretch and active contraction resulted in significantly different EMG distribution in the proximal-distal direction (4.58 ± 0.24 vs. 4.45 ± 0.11, *p* = 0.005). The *j*- coordinate of the CoG during passive stretch was larger when compared with the active contraction, which indicated a more distal location of its CoG.

## Discussion

There were three main findings drawn from this HD-sEMG study: (1) the spastic group showed velocity-dependent heterogeneous activation during passive stretch; (2) the spastic group showed higher activation intensity as well as more lateral and proximal location of the activation center than the healthy group during active contraction; (3) the activation center of passive stretch was situated at a more distal location than the activation center of active contraction in the spastic group, and most of their activation areas did not overlap. The comprehensive knowledge of neuromuscular dysfunction associated with spasticity not only helps to understand its mechanism but also assists in adjusting targeted intervention and formulating personalized rehabilitation.

### Stroke-induced changes in activation intensity and distribution of BB

When compared to the healthy group, the spastic group exhibited a significant increase in the reflex EMG response to the rapid passive stretch, which reflected the presence of a hyperexcitable stretch reflex. Consistent with previous studies on inducing spasticity [20, 28, 31], the observed global BB activity in the current study was positively correlated with stretch velocity in spastic hemiparetic subjects. It can be explained by that the velocity-dependent increase in reflex excitability is mediated by deficient inhibitory input directed to the spinal cord after CNS lesions [17]. Although the selected velocity of 10˚/s was not deemed to have elicited the stretch reflex response, the spastic group also displayed a higher baseline EMG intensity than the healthy group, presumably due to the increased background activity at rest [32, 33]. The activation pattern of the muscle can be revealed by activation distribution [34], while the spatial distribution of spastic muscles during passive stretch has been rarely investigated. These findings serve to support our hypothesis that the involuntary muscle activity induced by spasticity is spatially heterogeneous, indicating the non-uniform recruitment of MUs in the presence of spasticity. The global BB activity analysis also showed that the activation intensity of the spastic group was significantly higher than that of the healthy group when performing 20%MVC and 50%MVC active contraction. This is attributable to the requirement that hemiparetic subjects need to recruit more high-threshold MUs to produce a given level of contraction due to the reduced firing rate of MUs [13, 35]. This difference was not observed in the high-level contraction of 80%MVC, which may be related to the inability of hemiparetic subjects to effectively recruit more high-threshold MUs. In our spatial distribution analysis of BB activity during active activation, we found that the healthy group had two EMG peaks, while the spastic group had only one EMG peak. Studies have confirmed that the innervation zone (IZ) located at a midway point between the origin and insertion of the BB has the smallest EMG intensity [13, 36, 37], which may be involved in the separation between EMG peaks in the healthy group. We also found that the center of spastic BB activity was positioned more lateral and proximal than that of healthy BB activity. Recent evidence from the HD-sEMG study shows that the spatial heterogeneity of muscle activity is related to the spatial dependency of MUs control [38–40], and the healthy subjects mainly activate the short head of the BB during active contraction at 90˚ elbow flexion [41]. Therefore, these results suggested that the spatial recruitment pattern of MUs had changed in spastic hemiparetic subjects.

### Relationship between activation distribution of spastic BB during passive stretch and active contraction

Given that spasticity is related to voluntary movement, we compared the spatial distribution of spastic BB activity during passive stretch and active contraction in hemiparetic subjects. Our results revealed a low degree of overlapping (14.1% ± 13.4) between identified activation areas of the spastic BB during passive stretch and active contraction, while their respective area overlapping between different stretch velocities of passive stretch and between different contraction levels of active contraction were all above 80%. These results suggested that the MUs recruitment patterns of spastic BB were similar within the subtasks of passive stretch or active contraction, whereas the patterns were different between the passive stretch and active contraction. Previous studies have shown that upper limb function can predict upper limb spasticity and patients with poor motor recovery may exhibit increased muscle tone [3, 32]. This may be due to complex interactions between pathological neuromuscular activation resulting from loss of descending corticospinal pathways to spinal motoneurons, yet remains poorly understood. Spasticity is an excessive spinal reflex response during passive stretch, and the motoneuron recruitment is also compressed during active contraction due to altered cortical activation [12, 40]. In our study, we aim to explore the spatial relationship between involuntary and voluntary muscle activity affected by descending pathway damage after stroke. We provide new spatial evidence for an inverse relationship between muscle tone and motor function, consistent with earlier studies. Davidowitz et al. and Turpin et al. also found that the modulation of the stretch reflex was associated with the degree of functional impairment caused by stroke [14, 15]. Therefore, it can be speculated that the affected MUs may not be inhibited during involuntary spastic BB activity and may not be recruited during voluntary spastic BB activity, resulting in opposite MU recruitment patterns. Since the number of MUs is certain, the more MUs are affected, the fewer MUs can be recruited voluntarily, and vice versa. The relationship between passive stretch and active activation is not only manifested in the spatial complementarity of activation areas, but also in the more distal distribution of passive stretch than active activation. It has been reported that the distal muscles of stroke survivors are more severely injured than the proximal muscles [42–44], and the proximal and distal regions within a muscle have different activation patterns [45, 46]. Muscles are innervated by neural signals from the motoneuron pool [24], and studies have shown that there are some maladaptive changes in the spinal cord after CNS lesion [47, 48]. Therefore, the neural drive to some regions of the spastic BB may be cortical-independent during passive stretch, leading to unwanted recruitment of distal MUs.

Several limitations of this study need to be acknowledged. Firstly, this study includes a relatively small sample size. Secondly, the age distribution between the healthy group and the spastic group is not exactly matched. Our findings revealed that only the spastic group showed heterogeneous activation induced by spasticity during passive stretch, supporting that this age difference did not significantly impact the results. Since the different ages may have different motor control capabilities, the impact of age might exist in the active contraction. However, the impact of age is likely minimal given that the comparison between passive stretch and active contraction is performed within the spastic group. These findings demonstrate significant changes in the neuromuscular characteristics of spastic hemiparetic subjects. More age-matched subjects should be recruited for further investigation to confirm the significance of our findings. Thirdly, the HD-EMG decomposition technique would help to provide direct evidence regarding the underlying mechanism of spasticity on the MU level. From this perspective, the findings in our current work can provide guidance for HD-EMG decomposition, and more in-depth discussions should be conducted in conjunction with the decomposition technique in the future. Finally, a cross-sectional study could not dynamically reflect the adaptive changes of the spinal cord during recovery after stroke. Recording from the longitudinal study could have provided additional information on the different responses of spastic muscles during passive stretch and active contraction.

## Conclusions

This study explored the neuromuscular changes in spastic hemiparetic subjects using the HD-sEMG technique and found a velocity-dependent heterogeneous activation response to passive stretch. Furthermore, our findings add to new evidence implicating a spatial relationship of spastic BB activity between passive stretch and active contraction, which has important implications for understanding the mechanism of spasticity.

## Data Availability

The datasets used and/or analysed during the current study are available from the corresponding author on reasonable request.

## Abbreviations

%HD-sEMG: high-density surface electromyography
BB: biceps brachii
MUs: motor units
CNS: central nervous system
ADL: activity of daily living
QoL: quality of life
MAS: modified Ashworth scale
MMT: manual muscle test
MVC: maximum voluntary contraction
RMS: root mean square
CoG: root mean square
ANOVA: root mean square
IZ: innervation zone
